# Dietary fatty acids differentially impact phagocytosis, inflammatory gene expression, and mitochondrial respiration in microglial and neuronal cell models

**DOI:** 10.3389/fncel.2023.1227241

**Published:** 2023-08-10

**Authors:** Michael J. Butler, Sabrina E. Mackey-Alfonso, Nashali Massa, Kedryn K. Baskin, Ruth M. Barrientos

**Affiliations:** ^1^Institute for Behavioral Medicine Research, The Ohio State University, Columbus, OH, United States; ^2^Department of Psychiatry and Behavioral Health, The Ohio State University, Columbus, OH, United States; ^3^Medical Scientist Training Program, The Ohio State University, Columbus, OH, United States; ^4^Department of Physiology and Cell Biology, Dorothy M. Davis Heart and Lung Research Institute, The Ohio State University, Columbus, OH, United States; ^5^Department of Neuroscience, The Ohio State University, Columbus, OH, United States; ^6^Chronic Brain Injury Program, The Ohio State University, Columbus, OH, United States

**Keywords:** inflammation, lipids, nutrition, metabolism, synapse

## Abstract

The consumption of diets high in saturated fatty acids and/or refined carbohydrates are associated with neuroinflammation, cognitive dysfunction, and neurodegenerative disease. In contrast, diets high in polyunsaturated fatty acids are associated with anti-inflammatory and neuroprotective effects. We have previously shown that high fat diet (HFD) consumption increases saturated fatty acids and decreases polyunsaturated fatty acids in the hippocampus. We have further shown that HFD elicits exaggerated neuroinflammation and reduced synaptic elements, and results in robust memory deficits in aged rats. Here, we examined the impact of palmitate, an abundant dietary saturated fat, on a variety of cellular responses in BV2 microglia and HippoE-14 neurons, and the extent to which the omega-3 fatty acid, docosahexaenoic acid (DHA), would buffer against these responses. Our data demonstrate that DHA pretreatment prevents or partially attenuates palmitate-induced alterations in proinflammatory, endoplasmic reticulum stress, and mitochondrial damage-associated gene expression in both cell types. Furthermore, we show that synaptoneurosomes isolated from aged, HFD-fed mice are engulfed by BV2 microglia at a faster rate than synaptoneurosomes isolated from aged, chow-fed mice, suggesting HFD alters signaling at synapses to hasten their engulfment by microglia. Consistent with this notion, we found modest increases in complement proteins and a decrease in CD47 protein expression on synaptoneurosomes isolated from the hippocampus of aged, HFD-fed mice. Interestingly, palmitate reduced BV2 microglial phagocytosis, but only of synaptoneurosomes isolated from chow-fed mice, an effect that was prevented by DHA pretreatment. Lastly, we measured the impact of palmitate and DHA on mitochondrial function in both microglial and neuronal cell models using the Seahorse XFe96 Analyzer. These data indicate that DHA pretreatment does not mitigate palmitate-induced reductions in mitochondrial respiration in BV2 microglia and HippoE-14 neurons, suggesting DHA may be acting downstream of mitochondrial function to exert its protective effects. Together, this study provides evidence that DHA can ameliorate the negative impact of palmitate on a variety of cellular functions in microglia- and neuron-like cells.

## 1. Introduction

It is well established that consumption of diets high in saturated fatty acids (SFAs) and/or refined carbohydrates lacking dietary fiber lead to an increase in circulating free fatty acids and weight gain (Ferreira et al., 2004; Sumiyoshi et al., 2006; Ferramosca et al., 2014; Liu et al., 2015; Spadaro et al., 2015). These diets are also associated with neuroinflammation, cognitive dysfunction, and neurodegenerative disease (Miller and Spencer, 2014). We have previously shown that consumption of a short-term high fat diet (HFD) is sufficient to increase the levels of hippocampal SFAs and evoke a robust proinflammatory response in the hippocampus that leads to impairments in long-term memory in aged rats (Spencer et al., 2017; Butler et al., 2020, 2023). Conversely, consumption of diets enriched with polyunsaturated fatty acids (PUFAs) have been shown to reduce the brain’s response to an inflammatory stimulus and promote a return to homeostasis (Layé, 2010; Labrousse et al., 2012; Layé et al., 2018). In line with this observation, we recently demonstrated that dietary docosahexaenoic acid (DHA), an omega-3 PUFA, can ameliorate the increase in proinflammatory gene expression and cognitive impairment elicited by a refined carbohydrate diet in aged rats (Butler et al., 2021). That said, the cellular mechanisms mediating the effects of these different fatty acids on neuroinflammation are less clear.

Microglia are myeloid cells derived from erythromyeloid progenitors in the yolk sac and enter the nervous tissue early during embryonic development and serve as the resident innate immune cell of the brain parenchyma (Kierdorf et al., 2013). This, coupled with the fact they are also highly sensitive to fatty acid modulation (Nadjar, 2018; Leyrolle et al., 2019; Chausse et al., 2021), makes them a leading candidate for mediating diet-induced neuroinflammation. Indeed, we have shown that hippocampal microglia isolated from HFD-fed rats have increased proinflammatory transcripts relative to microglia isolated from chow-fed rats (Butler et al., 2020). Moreover, *ex vivo* treatment of isolated microglia with the SFA palmitate (PA), the most abundant dietary SFA, mimics the effects of HFD on microglial reactivity and the proinflammatory response (Butler et al., 2020). In the same study, we showed that HFD consumption decreased the concentration of PUFAs in the hippocampus and that aging was also associated with a reduction in DHA concentration in the hippocampus (Butler et al., 2020). It is known that DHA can exert its inflammation-resolving effects via direct action on microglia in response to a variety of brain insults, including neurodegenerative disease, traumatic brain injury, and the endotoxin lipopolysaccharide (LPS) (De Smedt-Peyrusse et al., 2008; Salem et al., 2015; Butt et al., 2017). However, the ability of PUFAs, such as DHA, to attenuate SFA-induced microglial reactivity is unknown.

In addition to microglia, neurons are also susceptible to injury due to direct and indirect lipid modulation. Previous work has shown that direct treatment with PA can induce an increase in endoplasmic reticulum (ER) stress and a decrease in cell viability in cultured hypothalamic neurons (Tse and Belsham, 2018). In the hippocampus, HFD and PA have been shown to impair long-term potentiation and contribute to the development of Alzheimer’s disease (AD) neuropathology (Contreras et al., 2017; Desale and Chinnathambi, 2020). Indirectly, synaptic integrity can be damaged due to excessive synaptic engulfment, another hallmark of AD, by reactive microglia in HFD-fed mice (Hao et al., 2016). However, the potential therapeutic effects of DHA on neuronal health in the context of SFA-induced injury is unknown.

Thus, in the current study, we used immortalized BV2 microglia to investigate the impact of PA and DHA treatment on microglial gene expression, phagocytic capacity, and mitochondrial function. We hypothesized that PA treatment would alter the expression of genes associated with inflammation, ER stress, mitochondrial homeostasis, and autophagy, as well as alter phagocytic function and mitochondrial respiration in BV2 microglia, and that DHA pretreatment would prevent or attenuate these changes. Furthermore, we used immortalized HippoE-14 neurons, a murine hippocampal neuronal cell line, to investigate the role of PA and DHA in neuronal gene expression and mitochondrial function. Similar to microglia, we hypothesized that DHA would mitigate PA-induced changes in expression of genes associated with inflammation, ER stress, mitochondrial homeostasis, and autophagy, and changes in mitochondrial respiration in HippoE-14 neurons.

## 2. Materials and methods

### 2.1. Cell culture and reagents

BV2 microglia were a kind gift from Dr. Jonathan Godbout and HippoE-14 neurons were purchased from Cedarlane Labs (CLU198, under MTAIN-051533). Both cell types were cultured in high-glucose Dulbecco’s modified Eagle’s medium (DMEM) supplemented with 10% FBS and 1% penicillin/streptomycin and maintained at 37°C and 5% CO_2_ atmosphere unless otherwise noted. In all experiments, a 1,000x concentration of sodium palmitate (P9767, Sigma) was dissolved in molecular biology grade water heated to 70°C as previously described (Ye et al., 2016; Tse and Belsham, 2018). For all experiments, a final concentration of 100 μM PA was used, which is consistent with previous studies that treated microglia, astrocytes, or neurons with palmitate (Listenberger et al., 2001; Wang et al., 2012; Duffy et al., 2015; Frago et al., 2017; Hidalgo-Lanussa et al., 2017; Sergi et al., 2018; Tse and Belsham, 2018; Yanguas-Casás et al., 2018) and is consistent with a physiological range of plasma palmitic acid levels (Abdelmagid et al., 2015). DHA (D2534, Sigma) was dissolved in 100% molecular biology grade ethanol at 1,000x concentration and diluted to working concentration of 30 μM, which is consistent with previous concentrations used with BV2 microglia (De Smedt-Peyrusse et al., 2008).

### 2.2. Effects of DHA and PA on gene expression BV2 microglia and HippoE-14 neurons

BV2 microglia and HippoE-14 neurons were independently cultured in DMEM as described above in 6-well plates and treated with either 30 μM DHA or vehicle for 24 h. Cells were then washed with media and treated with 100 μM PA or vehicle (*n* = 6 wells/group) for 6 h. Treatments were counterbalanced across plates and plates were run on two different days. Following treatment, cells were lysed with TRI-reagent (Sigma) and stored at −80°C until processed for qPCR.

### 2.3. RNA extraction and quantitative PCR (qPCR) measurement of gene expression

Ribonucleic acid from lysed cells was extracted as previously described (Butler et al., 2021, 2023). Briefly, after cells were lysed with TRI-reagent, chloroform was added to each sample at a 1:5 concentration and the samples were spun at 12,000 rcf for 15 min at 4°C. Next, the clear aqueous phase present after centrifugation was collected and used for RNA precipitation with molecular biology grade isopropanol at room temperature for 10 min, followed by a 10 min centrifugation at 12,000 rcf at 4°C. The resulting RNA pellet was washed in 75% molecular biology grade ethanol and spun again at 7,500 rcf for 5 min at 4°C. Finally, the supernatant was removed and the resulting RNA pellet was resuspended in 40 mL of DNase- and RNase-free water. RNA concentration was measured by nanodrop. Following RNA measurement, 1 μg of RNA was used to synthesize cDNA using qScript cDNA SuperMix (95048-100; Quantabio) according to kit instructions. cDNA was diluted 1:4 for qPCR. A detailed description of the PCR amplification protocol has been published previously (Frank et al., 2006). Primer sequences were designed using the Operon Oligo Analysis Tool^1^ and tested for sequence specificity using the Basic Local Alignment Search Tool at NCBI (Altschul et al., 1997). Primers were obtained from Invitrogen for the following genes: interleukin-1beta (*Il1b*), interleukin-6 (*Il6*), tumor necrosis factor alpha (*Tnf*), toll-like receptor 4 (*Tlr4*), C/EBP homologous protein (*Chop*), G protein-coupled receptor 78 (*Gpr78*), mitochondrial E3 ubiquitin protein ligase 1 (*Mul1*), PTEN-induced kinase 1 (*Pink1*), autophagy related gene 5 (*Atg5*), and autophagy related gene 12 (*Atg12*). Primer specificity was verified by melt curve analysis. All primers were designed to exclude amplification of genomic DNA. Primer sequences are listed in Table 1. PCR amplification of cDNA was performed using the Quantitect SYBR Green PCR Kit (Qiagen, Valencia, CA). Formation of PCR product was monitored in real time over 40 cycles using the QuantStudio 3 PCR System (Applied Biosystems, Waltham, MA, USA). Relative gene expression was determined by the ΔΔCT method of qPCR analysis normalized to β-Actin (*Actb*). For both cell lines, there were no group differences detected in Actb CT values [BV2: *F*(1,20) = 1.345, *p* = 0.25; HippoE-14: *F*(1,20) = 0.054, *p* = 0.82]. The mean ΔΔCT of the vehicle-vehicle group was used as the calibrator to calculate LOG2 fold-changes (control group set to 0) in mRNA concentrations as we have done previously (Butler et al., 2020, 2021). With this method, fold changes lower than 1 are converted to a negative value and fold changes greater than 1 are converted to a positive value.

**TABLE 1:** Primer sequences used for qPCR analysis.

Genes	Species	Forward	Reverse
*Il1b*	Mouse	TGCTGTCGGACCCATATGAG	GAAAAGAGTTGTGCAATGGC
*Il6*	Mouse	CCCTCACACTCAGATCATCT	GCTGGATTTATCCAGGTGTG
*Tnf*	Mouse	TATGAGGATCTGCAGGAG	GCGACAAGCAACCAAAGATG
*Tlr4*	Mouse	GCTTGCCAATCCCTTCTATG	GTCATCGAAGGAGCTGTGC
*Chop*	Mouse	GGACAGCTGCACACACTTGG	TGAATCAGTCCTTTGCCCCT
*Gpr78*	Mouse	TCGTGCGTGACATCAAAGAG	ATCCACACTCTCCAGCTGCA
*Pink1*	Mouse	TATGGTACTCCAGAAGACCA	TGTCTTTGAGATCCATGCCG
*Mul1*	Mouse	AGTCCAGAGAAACTTCCTGG	CAGGGTCAAGAGTAGTGAAG
*Atg5*	Mouse	TTCTTCTCTCCCTCTCTCTTATCC	CTCTCGCTGGAGCAGTGAC
*Atg12*	Mouse	GTAGTTCGGTTCCACACCATC	CATGCCTGGGATTTGCAGT
*Actb*	Mouse	CATGCCTGGGATTTGCAGT	GGATTCCATACCCAAGAAGG

### 2.4. Synaptoneurosome isolation and conjugation to pHrodo

Eighteen-month-old C57BL/6 male mice obtained from the National Institute on Aging rodent colony maintained by Charles River were fed either a standard chow (Teklad Diets, TD.8640; energy density of 3.0 kcal/g; 32% calories from protein, 54% from carbohydrates, and 14% from fat), or a HFD (Envigo, TD.06414; energy density of 5.0 kcal/g; 18% calories from protein, 22% from carbohydrates, and 60% from fat) for 3 days. On the 4th day, mice received a lethal dose of sodium pentobarbital (Fatal Plus) and then were transcardially perfused with ice-cold saline (0.9%) to remove any circulating immune leukocytes from the CNS vasculature. Following perfusion, brains were quickly extracted, placed on an ice-cold glass plate and hippocampi were dissected. This experiment was conducted in accordance with protocols approved by the Ohio State University Animal Care and Use Committee. Every effort was made to minimize the number of animals used and their suffering. Synaptoneurosomes were isolated from the hippocampus using the Syn-PER extraction buffer (87,793, Fischer Scientific, Waltham, MA, USA) according to product instructions. After total protein quantification using a Bradford assay, 300 μg of synaptoneurosome protein from each group were reconstituted in sodium bicarbonate and incubated with 1 μL of pHrodo (P35371, Fischer Scientific, Waltham, MA, USA) for 90 min at room temperature, protected from light, with gentle agitation. This thiol-reactive pH-sensitive dye emits its fluorescent signal when the environment becomes more acidic, such as when the bioconjugates it is bound to are engulfed by cells. Following the 90 min conjugation, synaptoneurosomes were spun at 19,000 rcf for 2 min and the supernatant was removed. The pellet was washed with 1x DPBS and spun at 19,000 rcf for 2 min. This was repeated 7 times to ensure any unbound pHrodo was removed. Synaptoneurosomes were reconstituted in 200 uL of 1x DPBS with 5% DMSO and stored at −20°C until use.

### 2.5. Live cell imaging and phagocytosis assay

For phagocytosis experiments, BV2 cells were incubated in phenol red-free high glucose DMEM supplemented with 10% FBS and 1% penicillin/streptomycin. In the first experiment, untreated BV2 cells were transferred to a 24-well plate (100 k cells/well) and allowed to adhere overnight. The following morning, cells were washed in sterile 1x DPBS and then incubated for 5 min with Hoechst stain for nuclear labeling of live cells. Cells were then washed twice with sterile 1x DPBS and phenol red-free DMEM containing 2 μg of pHrodo-conjugated synaptoneurosome protein isolated from either chow- or HFD-fed mice (*n* = 7 wells/group) were added to the wells. In the second experiment, cells were transferred to 24-well plates (100 k cells/well) and immediately treated with 30 μM DHA or vehicle for 24 h prior to a 6 h treatment of 100 μM PA or vehicle (*n* = 6–9 wells/group). Following the second treatment, cells were stained with Hoechst as described above and phenol red-free DMEM containing 2 μg of synaptoneurosome protein isolated from either chow or HFD-fed aged mice were added to the wells.

For both experiments, the 24-well plate was placed into a Cytation 1 live cell imager and maintained at 37°C and 5% CO2 atmosphere for the duration of the assay. For the assay, two images were automatically captured in each well every 15 min for the first hour and then once every hour for the following 3 h. Hoechst nuclear staining was used to quantify total number of cells and pHrodo-positive cells were automatically quantified and expressed as a percentage of total cells. For all experiments, conditions were counterbalanced across multiple plates and different plates were run on different days.

### 2.6. Western blot

To measure expression levels of classical complement cascade-related proteins on synapses, western blot analyses were run on synaptoneurosomes isolated from chow- or HFD-fed aged mice (*n* = 6/group), as described above. An equal amount of total protein (15 μg) from each sample was loaded into each lane. The NuPAGE Bis-Tris (10 well, 4–12%, 1.5 mm) gel electrophoresis system was used under reducing conditions (Life Technologies). The iBlot dry-blotting system (Life Technologies) was used to electrophoretically transfer gels to nitrocellulose membranes. Odyssey TBS blocking solution (Li-Cor) was used to prevent non-specific protein binding (1 h at RT). Primary antibodies, in Odyssey blocking solution containing 0.2% Tween20 (overnight at 4°C), were: C1q (1:1,000; sc-58920; Santa Cruz), C3 (1:200; ab200999; Abcam), and CD47 (1:1,000; PA5-109497; Thermo Fisher). Anti-GAPDH (1:50,000; 8,245; Abcam) was used as an internal loading control. There was no difference in GAPDH concentration between conditions [*t*(10) = 0.585, *p* = 0.57]. Blots were washed 4 × 5 min with TBS + 0.1% Tween20 and then probed with the appropriate fluorescent secondary antibody (1:5,000; Li-COR) for 1 h at RT. The Odyssey Infrared Imaging System (LI-COR Biosciences) was used to image membranes and Empiria Studio v1.3 software was used for quantification of bands. Each protein band was normalized to its respective loading control and then analyzed as percentage of control.

### 2.7. Mitochondrial stress test in BV2 and HippoE-14

To investigate the impact of fatty acids on mitochondrial function, we used the Seahorse XFe96 Analyzer. BV2 and HippoE-14 cells were independently incubated in phenol red-free high glucose DMEM supplemented with 10% FBS and 1% penicillin/streptomycin. Cells were transferred to a 96-well Seahorse microplate (7 k/well) and treated with 30 mM DHA or vehicle for 24 h prior to a 6 h treatment of 100 μM PA or vehicle (n = 15 wells/group). Following PA treatment, cells were washed in sterile 1x DPBS and then incubated in serum-free Agilent Seahorse DMEM containing 5 μM glucose, 1 μM sodium pyruvate, and 1 μM L-glutamine in a CO_2_-free incubator maintained at 37°C for 1 h. To quantify oxygen consumption rate (OCR), which is an indicator of mitochondrial respiration, three baseline OCR measurements were taken followed by sequential addition of oligomycin (1.5 μM well concentration), carbonyl cyanide-4 (trifluoromethoxy) phenylhydrazone (FCCP; 1.0 μM well concentration), and a combination of rotenone and antimycin A (0.5 μM well concentration). Following the assay, cells were fixed with 4% paraformaldehyde and nuclei were stained with DAPI. Total cells were automatically counted by a Cytation 1 imager. The raw OCR data from each well were normalized to total cell counts from the corresponding well. Total cell counts did not differ across treatments. Both raw and normalized data are presented in the “Results” section.

### 2.8. Data analysis

All data are presented as means ± the standard error of the means (SEM). Statistical analyses were computed using GraphPad Prism version 9. Outliers, as determined by Grubb’s outlier test, were eliminated prior to statistical analyses. Due to our 2 × 2 factorial design, two-way ANOVAs (PA × DHA) were run for each gene in qPCR experiments. For phagocytosis experiments, statistical analyses were performed on the 4 h time point (cumulative phagocytosis). In the first phagocytosis experiment, there were only two groups (aged-chow vs. aged-HFD synaptoneurosomes), so a Student’s *t*-test was used. In the second phagocytosis experiment, in which we treated BV2 cells with fatty acids prior to the assay, a two-way (2 × 4) ANOVA was used. For Seahorse XFe96 experiments, the normalized OCR from the first time point of each phase of the assay was analyzed with a two-way ANOVA. For all two-way ANOVAs, in the case of significant interactions, Turkey’s multiple comparisons *post-hoc* tests were run. For all statistical tests, the threshold for significance was set as α = 0.05. Only significant *F*-values were reported in the “Results” section.

## 3. Results

### 3.1. DHA pretreatment prevents PA-induced alterations in genes associated with inflammation, ER stress, mitochondrial homeostasis, and autophagy in BV2 microglia

**FIGURE 1:**
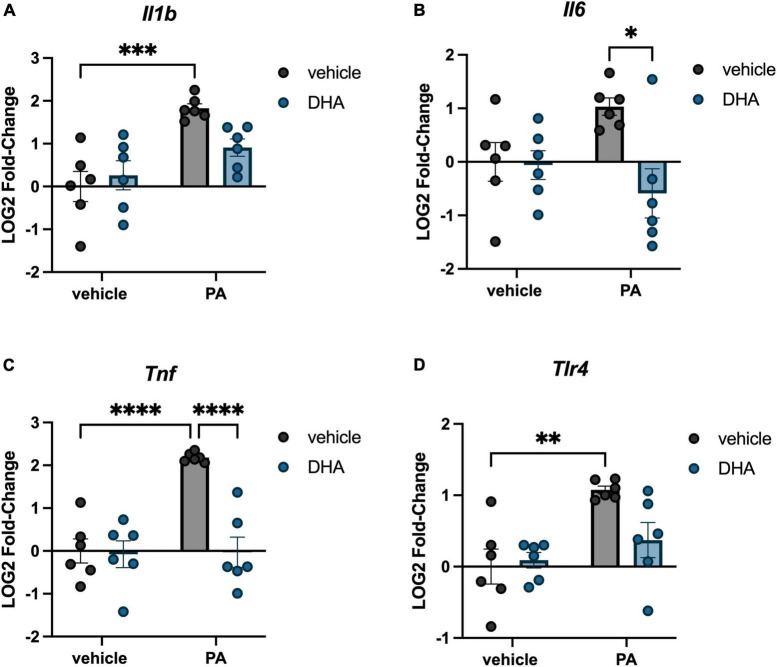
DHA attenuates PA-induced increases in inflammatory gene expression in BV2 microglia. Effects of DHA and PA on **(A)** *Il1b*, **(B)** *Il6*, **(C)** *Tnf*, and **(D)** *Tlr4* gene expression. *p < 0.05, ^**^p < 0.01, ^***^p < 0.001, and ^****^p < 0.0001.

**FIGURE 2:**
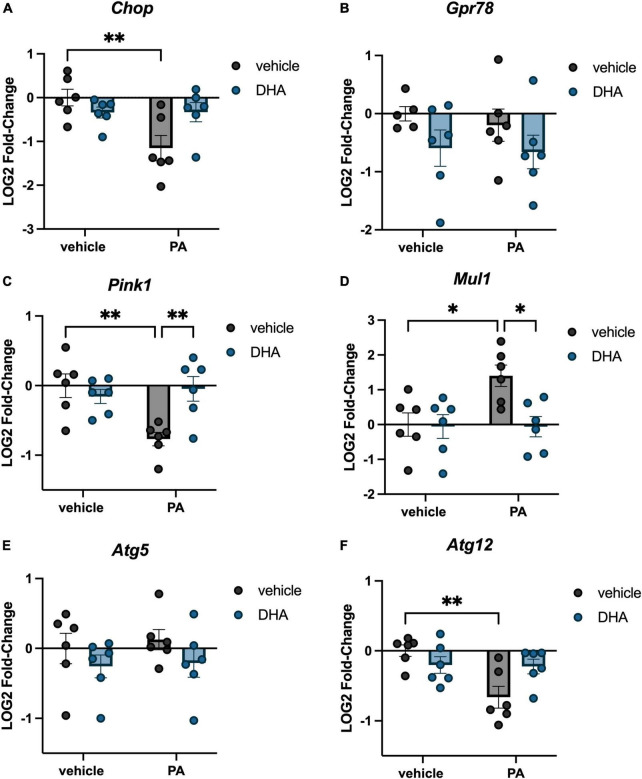
DHA attenuates PA-induced changes in ER stress, mitochondrial homeostasis, and autophagy gene expression in BV2 microglia. Effects of DHA and PA on **(A)** *Chop*, **(B)** *Gpr78*, **(C)** *Pink1*, and **(D)** *Mul1*, **(E)** *Atg5*, and **(F)** *Atg12* gene expression. *p < 0.05 and ^**^p < 0.01.

We investigated the impact of PA on gene expression in BV2 microglia and the extent to which DHA pretreatment would ameliorate any of these changes. For genes associated with inflammation, there were significant PA x DHA interactions for *Il1b* [*F*(1,20) = 4.814, *p* < 0.05; Figure 1A], *Il6* [*F*(1,20) = 5.546, *p* < 0.05; Figure 1B], *Tnf* [*F*(1,20) = 14.980, *p* < 0.001; Figure 1C], and *Tlr4* [*F*(1,20) = 4.661, *p* < 0.05; Figure 1D]. *Post hoc* analyses indicated that PA treatment significantly increased the expression of these genes relative to vehicle-treated controls (*p* < 0.01). DHA treatment alone did not alter gene expression compared to vehicle-treated controls, and importantly, DHA + PA treatment blunted the PA-evoked increase, as gene expression did not differ from vehicle controls; however, this group was also not statistically different from the PA group for *Il1b* and *Tlr4* expression. Gene expression for the ER stress gene *Chop* resulted in a similar PA × DHA interaction [*F*(1,20) = 7.361, *p* < 0.05; Figure 2A], with PA significantly decreasing gene expression relative to vehicle controls (*p* < 0.01), and DHA + PA-treated cells were not different from vehicle- or PA-treated cells. *Gpr78*, another gene associated with ER stress, was not differentially expressed across groups (*p* > 0.05, Figure 2B).

In addition to inflammation- and ER stress-related genes, we investigated changes in genes associated with mitochondrial homeostasis. PINK1 is a mitochondrial kinase that promotes cell survival, particularly under conditions of oxidative stress and is integral in mediating mitochondria turnover (Springer and Kahle, 2011). MUL1 is an enzyme that localizes to the outer mitochondrial membrane, where it regulates mitochondrial morphology and apoptosis (Otomo et al., 2013). We found a significant PA × DHA interaction for both *Pink1* [*F*(1,20) = 9.672, *p* < 0.01; Figure 2C] and *Mul1* [*F*(1,20) = 4.833, *p* < 0.05; Figure 2D]. *Post hoc* analyses indicated that PA treatment significantly decreased *Pink1* expression (*p* < 0.01) and increased *Mul1* expression (*p* < 0.05) compared to vehicle controls. In both cases, DHA + PA treatment completely prevented these alterations in expression. Lastly, we examined two genes associated with autophagy, *Atg5* and *Atg12*, which form a protein complex and localize to the membrane of autophagic vesicles (Otomo et al., 2013). We observed no changes in *Atg5* (*p* > 0.05; Figure 2E), but we did observe a significant PA x DHA interaction with *Atg12* [*F*(1,20) = 7.464, *p* < 0.05; Figure 2F] in which PA treatment significantly decreased *Atg12* expression relative to vehicle-treated controls (*p* < 0.01), while DHA + PA-treated cells were not different from vehicle- or PA-treated cells.

### 3.2. BV2 microglia exposed to different fatty acids differentially engulf hippocampal synaptoneurosomes isolated from aged-HFD and aged-chow mice

**FIGURE 3:**
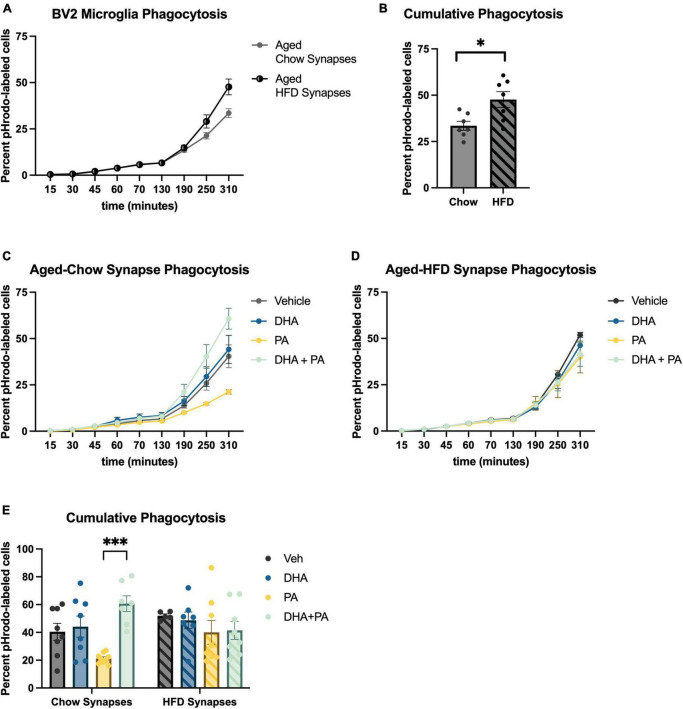
BV2 microglia differentially engulf hippocampal synaptoneurosomes isolated from aged-chow and aged-HFD mice. **(A)** Full time course of phagocytosis assay and **(B)** cumulative phagocytosis analysis of aged-chow vs. aged-HFD synaptoneurosomes. **(C)** Time course of phagocytosis analysis of aged-chow synaptoneurosomes and **(D)** aged-HFD synaptoneurosomes after BV2 microglia were treated with fatty acids. **(E)** Cumulative phagocytosis analysis of aged-chow and HFD synaptoneurosomes after BV2 microglia were treated with fatty acis. *p < 0.05 and ^***^p < 0.001.

The engulfment of weakened synaptic and other cellular debris is a critical function of microglia to maintain optimal synaptic transmission and plasticity (Cornell et al., 2022). However, during times of exaggerated neuroinflammation, these cells have been shown to over-engulf synapses resulting in impaired neuronal plasticity (Färber et al., 2009; Luchena et al., 2018; Bie et al., 2019; Wang et al., 2020; Wang and Li, 2021). Whether this increased phagocytosis is driven by increases in complement expression on synapses indicating greater “eat me” signals, or a dysregulation of microglia has not been determined. Also, the influence of fatty acids in this system has not been well-explored. To begin to disentangle these questions, we measured the phagocytosis of synaptoneurosomes, synaptic material containing pre- and post-synaptic terminals, by BV2 cells using live cell imaging *in vitro*. In the first experiment, untreated, naïve BV2 cells were incubated with synaptoneurosomes isolated from the hippocampus of either chow- or HFD-fed aged mice. Results indicated that synaptoneurosomes isolated from HFD-fed hippocampi were engulfed at a significantly faster rate than synaptoneurosomes isolated from chow-fed hippocampi [*t*(12) = 2.91, *p* < 0.05; Figures 3A, B]. In the second experiment, to examine how exposing microglia to fatty acids might further impact phagocytosis of synaptoneurosomes from the two different diet conditions, BV2 cells were treated with either PA or DHA + PA (as described in previous sections) prior to being incubated with synaptoneurosomes isolated from either chow-fed or HFD-fed aged hippocampi. Results indicate a significant fatty acid treatment × synapse condition interaction [*F*(3,52) = 3.66, *p* < 0.05; Figures 3C–E]. *Post hoc* analyses indicated that BV2 microglia exposed to PA alone suppressed phagocytic activity of synaptoneurosomes isolated from chow-fed aged hippocampi, though this was not statistically significant (Figure 3E). However, when cells were exposed to DHA + PA, phagocytic function was restored, as phagocytic activity was significantly higher than PA treatment alone (*p* < 0.001, Figure 3E). In contrast, there were no significant fatty acid-associated differences in phagocytic activity of BV2 cells exposed to aged-HFD synaptoneurosomes (*p* > 0.05; Figure 3E). To determine the extent to which immunological signals at the level of the synapse elicited by HFD might be driving this increased uptake by BV2 cells, we measured the expression of “eat me” complement proteins C1q and C3, and the “don’*t* eat me” protein CD47 in synaptoneurosomes isolated from either chow- or HFD-fed aged mice via Western blot. Results showed modest increases in C1q (Supplementary Figure 1A) and C3 (Supplementary Figure 1B) and a decrease in CD47 (Supplementary Figure 1C) in synaptoneurosomes isolated from aged HFD-fed mice, relative to those from aged chow-fed mice, but these alterations were not statistically significant (*p* > 0.05).

### 3.3. DHA pretreatment prevents PA-induced changes in genes associated with inflammation and ER stress in immortalized hippocampal neurons

**FIGURE 4:**
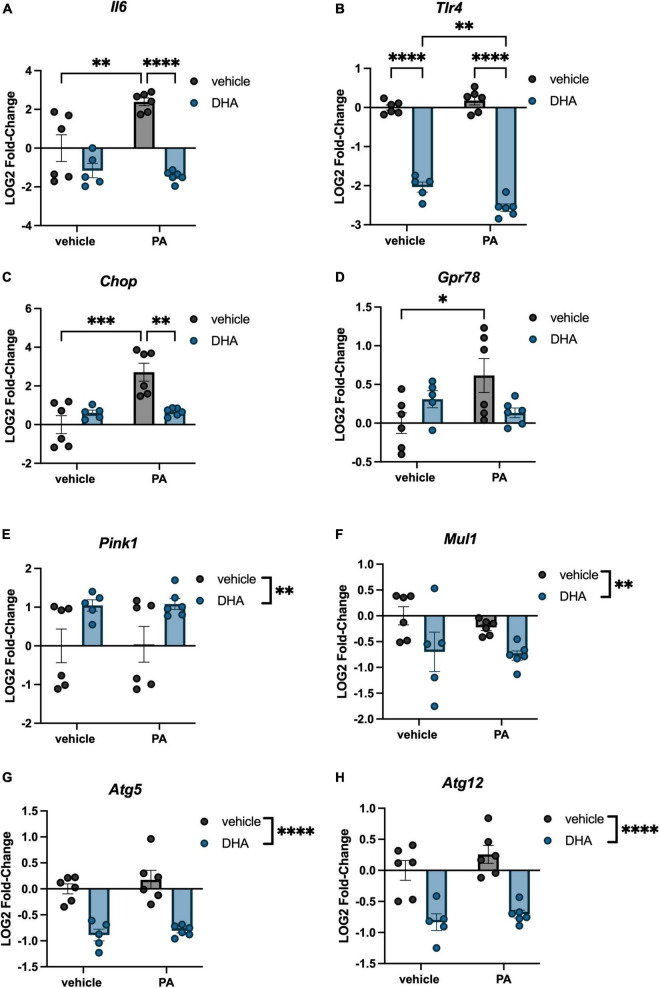
DHA and PA dysregulate inflammatory, ER stress, mitochondrial homeostasis, and autophagy gene expression in HippoE-14 neurons. Effects of DHA and PA on **(A)** *Il6* **(B)** *Tlr4*, **(C)** *Chop*, **(D)** *Gpr78*, **(E)** *Pink1*, and **(F)** *Mul1*, **(G)** *Atg5*, and **(H)** *Atg12* gene expression. *p < 0.05, ^**^p < 0.01, ^***^p < 0.001, and ^****^p < 0.0001.

Immortalized hippocampal neurons were treated with PA and DHA using the same concentrations and time course as was used with BV2 microglia, and expression of many of the same genes were assessed. For inflammation-related genes, we observed a significant PA × DHA interaction for *Il6* [*F*(1,19) = 10.50, *p* < 0.005; Figure 4A] and *Tlr4* [*F*(1,19) = 12.070, *p* < 0.005; Figure 4B]. *Post hoc* analysis of *Il6* indicated a PA-induced increase in expression compared to vehicle controls (*p* < 0.01), and a significant prevention of that increase with DHA + PA treatment (*p* < 0.001). *Post hoc* analysis of *Tlr4* showed that the DHA treatment alone significantly decreased expression relative to vehicle controls (*p* < 0.0001) and this decrease was even greater in DHA + PA-treated cells (*p* < 0.01).

Regarding ER stress genes, there was a significant DHA x PA interaction for both *Chop* [*F*(1,19) = 14.320, *p* < 0.005; Figure 4C] and *Gpr78* expression [*F*(1,19) = 7.302, *p* < 0.05; Figure 4D]. *Post hoc* analyses indicated significant PA-induced increases in *Chop* (*p* < 0.001) and *Gpr78* (*p* < 0.05) compared to vehicle controls. Importantly, DHA + PA treatment prevented this increase in *Chop* gene expression, as gene expression was not different from controls and was significantly different from PA-treated cells. For *Gpr78* expression, DHA + PA-treated cells were not different from vehicle- or PA-treated cells. For the mitophagy genes, we only observed a main effect of DHA to increase *Pink1* expression [*F*(1,19) = 9.996, *p* < 0.01; Figure 4E], and a main effect of DHA to decrease *Mul1* expression [*F*(1,19) = 7.764, *p* < 0.01; Figure 4F]. Lastly, we observed a main effect of DHA to decrease the expression of both autophagy-related genes *Atg5* [*F*(1,19) = 60.130, *p* < 0.0001; Figure 4G] and *Atg12* [*F*(1,19) = 46.980, *p* < 0.0001; Figure 4H].

### 3.4. DHA does not mitigate PA-induced reduction in mitochondrial respiration in BV2 microglia or HippoE-14 neurons

**FIGURE 5:**
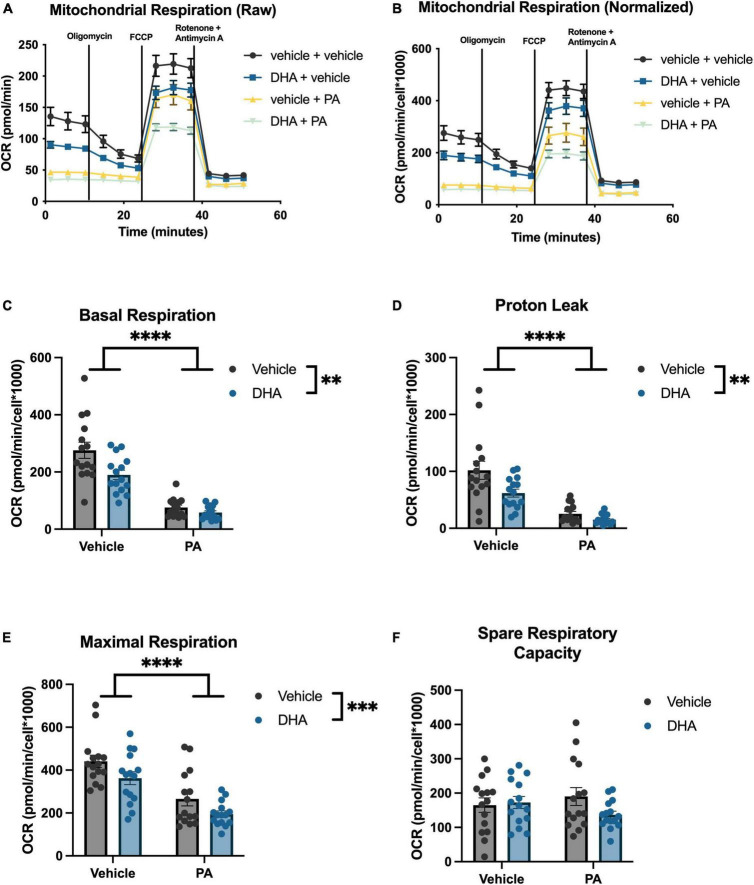
PA decreases mitochondrial respiration in BV2 microglia. Raw **(A)** and normalized **(B)** Seahorse XFe96 data. Effects of DHA and PA on **(C)** basal respiration, **(D)** proton leak, **(E)** maximal respiration, and **(F)** spare respiratory capacity of mitochondria. ^**^p < 0.01, ^***^p < 0.001, and ^****^p < 0.0001.

**FIGURE 6:**
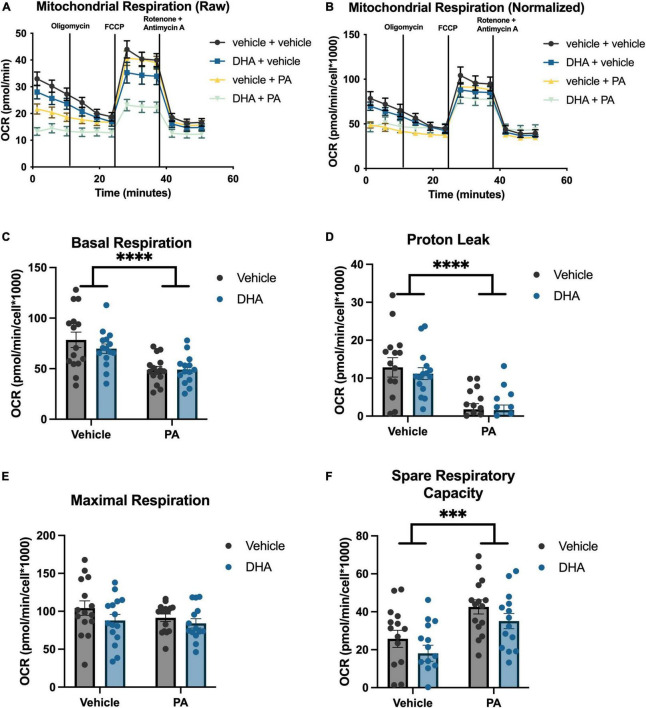
PA decreased mitochondrial respiration in HippoE-14 neurons. Raw **(A)** and normalized **(B)** Seahorse XFe96 data. Effects of DHA and PA on **(C)** basal respiration, **(D)** proton leak, **(E)** maximal respiration, and **(F)** spare respiratory capacity of mitochondria. ^***^p < 0.001 and ^****^p < 0.0001.

To understand the impact of DHA and/or PA on mitochondrial function in BV2 microglia and HippoE-14 neurons, we used the Seahorse XFe96 Analyzer to evaluate the oxygen consumption rate (OCR) of mitochondria. Data from each well was normalized to total cell number in each well and cell counts did not differ across groups. Data from BV2 microglia are presented in Figure 5, and data from HippoE-14 neurons are presented in Figure 6. Raw and normalized OCR for BV2 microglia are displayed in Figures 5A, B, respectively, but only normalized OCR data was used to analyze each phase of the assay. In BV2 microglia, there was a main effect of both PA and DHA to reduce basal respiration [PA: *F*(1,56) = 92.98, *p* < 0.0001; DHA: *F*(1,56) = 9.08, *p* < 0.005; Figure 5C], proton leak [PA: *F*(1,56) = 47.08, *p* < 0.0001; DHA: *F*(1,56) = 7.81, *p* < 0.01; Figure 5D], and maximal respiration [PA: *F*(1,56) = 24.88, *p* < 0.0001; DHA: *F*(1,56) = 7.35, *p* < 0.01; Figure 5E], and no impact of either treatment on spare respiratory capacity of mitochondria (Figure 5F). The raw and normalized OCR data for HippoE-14 neurons are presented in Figures 6A, B, respectively. In HippoE-14 neurons, there was a main effect of PA to reduce basal respiration [*F*(1,55) = 22.95, *p* < 0.0001; Figure 6C] and proton leak [*F*(1,55) = 32.24, *p* < 0.0001; Figure 6D]. There was no impact of either treatment on maximal respiration in HippoE-14 neurons (Figure 6E). However, there was a main effect of PA to increase the spare respiratory capacity of mitochondria in HippoE-14 neurons [*F*(1,55) = 16.79, *p* < 0.0001; Figure 6F]. DHA treatment did not impact any mitochondrial measure in HippoE-14 neurons.

## 4. Discussion

In the current study, we investigated the effects of both PA and DHA on gene expression, phagocytic function, and metabolic function in BV2 microglia, as well as gene expression and metabolic function in HippoE-14 neurons. Our data suggest that treatment of BV2 microglia with PA increased gene expression of multiple proinflammatory cytokines and altered the expression of several genes associated with ER stress, mitochondrial homeostasis, and autophagy. Importantly, DHA pretreatment normalized some of these alterations in PA-treated BV2 microglia. We also show, for the first time, that synaptoneurosomes isolated from HFD-fed mice were engulfed at a higher rate than synaptoneurosomes isolated from chow-fed mice. Moreover, PA treatment differentially impacted the uptake of chow- and HFD-synaptoneurosomes. Similar to its effect in BV2 microglia, PA treatment increased the expression of inflammation and ER stress genes in immortalized hippocampal neurons, but did not impact the expression of genes associated with mitochondrial homeostasis and autophagy. Interestingly, in HippoE-14 neurons, DHA treatment dramatically decreased inflammatory gene expression and normalized ER stress gene expression (*Chop*) in PA-treated cells. DHA also altered the expression of mitochondrial homeostasis and autophagy genes in HippoE-14 neurons, independent of PA treatment. Lastly, PA decreased mitochondrial respiration in both BV2 microglia and HippoE-14 neurons, and DHA did not mitigate these effects.

Our study demonstrated that 6 h PA treatment increased microglial expression of *Il1b*, *Il6*, *Tnf*, and *Tlr4* genes. These findings replicate previous work in primary and BV2 microglia that showed PA treatment increased transcripts associated with inflammation using a similar dose and time course (Hidalgo-Lanussa et al., 2017; Butler et al., 2020; Urso and Zhou, 2021). We extend that work by showing, for the first time, that pretreating microglial-like cells with DHA prevented (*Tnf*) or partially attenuated (*Il1b, Il6*, and *Tlr4*) these increases in inflammatory gene expression elicited by PA treatment. These anti-inflammatory effects of DHA on microglia extend previous reports showing DHA and DHA metabolites attenuate microglial inflammation in response to LPS, by showing a similar effect with SFA treatment (De Smedt-Peyrusse et al., 2008; Rey et al., 2016). Our findings also extend work done in primary astrocytes, in which DHA pretreatment mitigated PA-induced cytokine release (Gupta et al., 2012). In addition to triggering inflammatory responses in the brain, excess accumulation of PA and other SFAs can impair other aspects of cellular function (Tracy et al., 2013; Urso and Zhou, 2021). Thus, we demonstrated that PA treatment dysregulated BV2 microglial expression of several genes associated with ER stress (*Chop*), mitochondrial homeostasis (*Pink1 and Mul1*), and autophagy (*Atg12*) and that DHA pretreatment completely prevented (in the case of *Pink1 and Mul1*) or partially attenuated (*Atg12*) this dysregulation.

Our previous work in aged rats showed that HFD consumption decreases both pre- and postsynaptic elements in the hippocampus of aged rats, which is associated with deficits in hippocampal-dependent long-term memory (Butler et al., 2023). While the modulation of synapses via phagocytosis of synaptic elements is a beneficial and critical function of microglia throughout the lifespan, excessive synaptic engulfment is detrimental and has been reported to occur with greater frequency in aged brains (Duan et al., 2003; Dumitriu et al., 2010; Shi et al., 2015; Boros et al., 2019). This phagocytic process is susceptible to environmental modulation, including diet, and can have a significant impact on cognitive function (Hao et al., 2016; Cope et al., 2018; Wang and Li, 2021). Here, we show that BV2 microglia engulf hippocampal synaptoneurosomes isolated from HFD-fed aged mice at a faster rate than hippocampal synaptoneurosomes isolated from chow-fed aged mice. The use of an untreated immortalized microglial cell model, which is devoid of signals from the brain microenvironment and thus can only react to the synapses we expose them to, provides compelling evidence that HFD consumption directly modifies aged synapses in such a way that elicits greater engulfment by phagocytic cells.

It has previously been shown that increased expression of “eat me” complement proteins C1q and C3 at the synapse results in increased synaptic removal (Färber et al., 2009; Shi et al., 2015). Conversely, “don’*t* eat me” CD47 protein expression has been shown to negatively regulate phagocytosis (Zhang et al., 2015). However, the impact of diet on synaptic deposition of these proteins had not been investigated until now. We found that short-term HFD consumption evoked a slight increase in both C1q and C3, and a decrease in CD47 on synapses of aged mice. Although these HFD-evoked modulations from baseline were not statistically significant, the overall pattern of results suggests that HFD shifts these immunological signals in favor of greater phagocytosis of synapses. An important technical caveat concerns the low protein yield (only 15 μg of protein could be loaded) from mouse synaptoneurosomes isolated from the hippocampus, which likely contributed to some variability in our dataset. While we might have measured these proteins from whole brain samples, which would yield greater protein concentrations, we chose to limit analyses to hippocampal synaptoneurosomes as these are the most biologically relevant samples needed to address this question. Future work will examine a causal role of complement signaling in diet-induced synaptic engulfment and memory impairment.

Exposure of BV2 microglia to PA slowed their engulfment of synaptoneurosomes isolated from chow-fed hippocampi, an effect that was completely mitigated by DHA pretreatment. This PA-induced reduction in phagocytosis was unexpected, considering that HFD decreases synaptic elements *in vivo*, implying an increase in phagocytosis (Hao et al., 2016; Butler et al., 2023). Interestingly, while PA decreased engulfment of synaptoneurosomes isolated from chow-fed mice, it did not decrease engulfment of synaptoneurosomes isolated from HFD-fed mice. Again, this suggests a fundamental change to synapses, perhaps via complement and/or CD47 signaling, in HFD-fed aged mice that favors engulfment, even in the presence of a stimulus that reduces phagocytosis under basal conditions. Importantly, very little work has investigated the impact of PA and other SFAs on microglial phagocytosis. One study utilizing BV2 cells showed that a similar dose (125 μM) of PA potentiated LPS-induced phagocytosis, but did not have an effect on its own (Tracy et al., 2013). In primary microglia, treatment with 100 μM and 200 μM PA inhibited IFNγ-stimulated phagocytosis, but PA alone had no impact (Yanguas-Casás et al., 2018). Of note, while similar concentrations of PA were used in these studies, both studies treated cells for 24 h prior to measuring phagocytosis, as opposed to only 6 h in the current study. Moreover, these previous studies used antibody-coated fluorescent latex beads for their phagocytosis assays, which triggers Fc receptor-mediated phagocytosis (Ulvestad et al., 1994; Hughes et al., 2010). In the current study, the use of synaptic debris is not only more physiologically relevant, but also likely triggered a different mechanism of microglial phagocytosis, such as complement receptor signaling. Thus, any inconsistencies between these few studies could be explained by timing of SFA treatment and the triggering mechanism of phagocytosis. Regardless of the directionality of the PA effect, our study showed, for the first time, the ability of DHA to attenuate SFA-mediated effects on microglial phagocytosis. Of note, the increased phagocytosis effect of aged-HFD synaptoneurosomes in vehicle-treated cells observed in the first experiment did not reach significance in this second experiment, even though the same general pattern was observed. This discrepancy is likely due to inter-assay variability attributable to different batches of BV2 cells being used. Furthermore, while pHrodo-conjugated debris is reliably used to measure phagocytic capacity (Miksa et al., 2009; Neaga et al., 2013; Lenzo et al., 2016), observed differences could be due to a discrepancy in the degradation of engulfed material. Understanding these nuances will be the focus of future experiments.

In addition to the impact of fatty acids on microglia-like cells, we aimed to understand the direct impact of PA and DHA on neuron-like cells. Similar to BV2 cells, our results demonstrated that PA treatment increased *Il6* as well as the ER stress genes *Chop* and *Gpr78*. These data extend previous work showing PA increases ER stress genes in immortalized hypothalamic neurons (Tse and Belsham, 2018) by showing a similar result in immortalized hippocampal neurons. Furthermore, we show, to the best of our knowledge, the first evidence that DHA can prevent or partially attenuate SFA-induced ER stress in neuronal cells, which adds to the growing literature on DHA’s neuroprotective effects. While transcripts associated with mitochondrial homeostasis and autophagy in HippoE-14 neurons were not altered by PA, DHA did independently alter these transcripts, again highlighting the important role for DHA in neuronal cell function.

Because both cell types showed changes in transcripts associated with mitochondrial function as a result of fatty acid treatment, we directly measured mitochondrial respiration in both cell types. Indeed, treatment with PA impaired baseline mitochondrial respiration and proton leak-linked respiration in both BV2 and HippoE-14 cells. Moreover, PA treatment also inhibited maximal mitochondrial respiration in BV2 microglia, but not in HippoE-14 neurons. This finding suggests microglia, in the presence of high levels of SFAs, might be less capable of responding to a rapid increase in energy demand (simulated by FCCP treatment) than neurons, though future studies using primary cells are needed to confirm this. The finding that PA does not decrease maximal respiration and actually increases spare respiratory capacity, another measure of how well mitochondria respond to a rapid energy demand, in HippoE-14 neurons might suggest neuronal mitochondria have a greater ability than microglial mitochondria to functionally rebound following PA exposure. The magnitude of the PA effect on basal respiration was also greater in BV2 microglia, relative to HippoE-14 neurons, which further suggests microglia might be more vulnerable than neurons to SFA-induced injury. The effect of PA on mitochondrial respiration in BV2 microglia replicate previous work showing PA reduces metabolic activity in immortalized N9 microglia (Beaulieu et al., 2021), and closely mirrors the effect of LPS treatment in BV2 microglia, which is consistent with a proinflammatory phenotype (Chausse et al., 2020; Qiu et al., 2022) and impaired phagocytosis (Nadjar, 2018). Dysregulated cellular metabolism, specifically decreased mitochondrial respiration in both microglia and neurons, has been linked to neurodegenerative disease states, as has excessive intake of saturated fatty acids (Luchsinger et al., 2002; Barnard et al., 2014; Ruan et al., 2018; Agrawal and Jha, 2020). Likewise, the impact of SFAs on mitochondrial respiration could be a key mechanistic link between dietary saturated fat and neurodegenerative disease.

Docosahexaenoic acid treatment did not mitigate the decrease in mitochondrial respiration elicited by PA in BV2 microglia or HippoE-14 neurons. Because prior work suggests that cellular metabolism and inflammatory state are closely linked (Hu et al., 2020; Chausse et al., 2021), we hypothesized DHA would prevent any PA-induced changes in mitochondrial function. If PA-induced decreases in mitochondrial respiration are directly tied to changes in gene expression and phagocytosis (in BV2 microglia), perhaps DHA is acting downstream of these metabolic changes. In other words, DHA may not mitigate the metabolic effects of PA, but it can prevent the downstream changes in inflammatory state and phagocytic function in BV2 microglia and ER stress in HippoE-14 neurons via intracellular signaling or mitophagy-related processes. This hypothesis is in line with a previous study showing that DHA attenuated PA-induced cytokine release in astrocytes via a reduction in p38MAKP and p42/44 MAPK phosphorylation, independent of altering metabolic function (Gupta et al., 2012). Because we only measured mitophagy-associated gene expression (*Pink1*) in the current study and did not perform experiments that directly quantified total mitochondria and mitochondria turnover, we cannot rule out potential effects of DHA on these other aspects of mitochondrial health. Future experiments will attempt to disentangle the relationship between cellular metabolism and a variety of microglial and neuronal functions in the context of fatty acid signaling, including inflammation, phagocytosis, and mitophagy.

To conclude, our data provide the first evidence that DHA can ameliorate the impact of SFAs, specifically PA, on inflammatory, ER stress, mitochondrial dynamics, and autophagy gene expression and phagocytosis in microglia-like cells. Moreover, the finding that synaptoneurosomes isolated from HFD-fed aged mice are engulfed at a faster rate than those isolated from chow-fed aged mice provides exciting evidence that synapses from the aged brain are fundamentally altered by HFD in a way that facilitates their degradation via phagocytosis. The inclusion of neuron-like cells allows for some insight into brain cell-specific effects of these prominent fatty acids, as our data illustrate some intriguing differences between the two cell lines regarding gene expression and cell metabolism. However, the use of immortalized cell lines to investigate these cell-specific differences limits our ability to make definitive conclusions about how these effects play out *in vivo*, and thus, future work will use a combination of primary cells isolated from the brain and *in vivo* methods to extend the current findings.

## Data availability statement

The raw data supporting the conclusions of this article will be made available by the authors, without undue reservation.

## Ethics statement

The animal study was reviewed and approved by The Ohio State University Institutional Animal Care and Use Committee.

## Author contributions

MB: conceptualization, formal analysis, investigation, writing–original draft, and writing–review and editing. SM-A: formal analysis, investigation, and writing–review and editing. NM: investigation and writing–review and editing. KB: formal analysis and writing–review and editing. RB: conceptualization, formal analysis, and writing–review and editing. All authors contributed to the article and approved the submitted version.

## Footnotes

1
http://www.operon.com/technical/toolkit.aspx
