# Supplementary material for: Dietary fatty acids differentially impact phagocytosis, inflammatory gene expression, and mitochondrial respiration in microglial and neuronal cell models
Source: Front Cell Neurosci. 2023 Aug 10;17:1227241. doi: 10.3389/fncel.2023.1227241 (PMC10448530; doi:10.3389/fncel.2023.1227241)
Supplement: Supplementary file 1 [file Image_1.PDF]

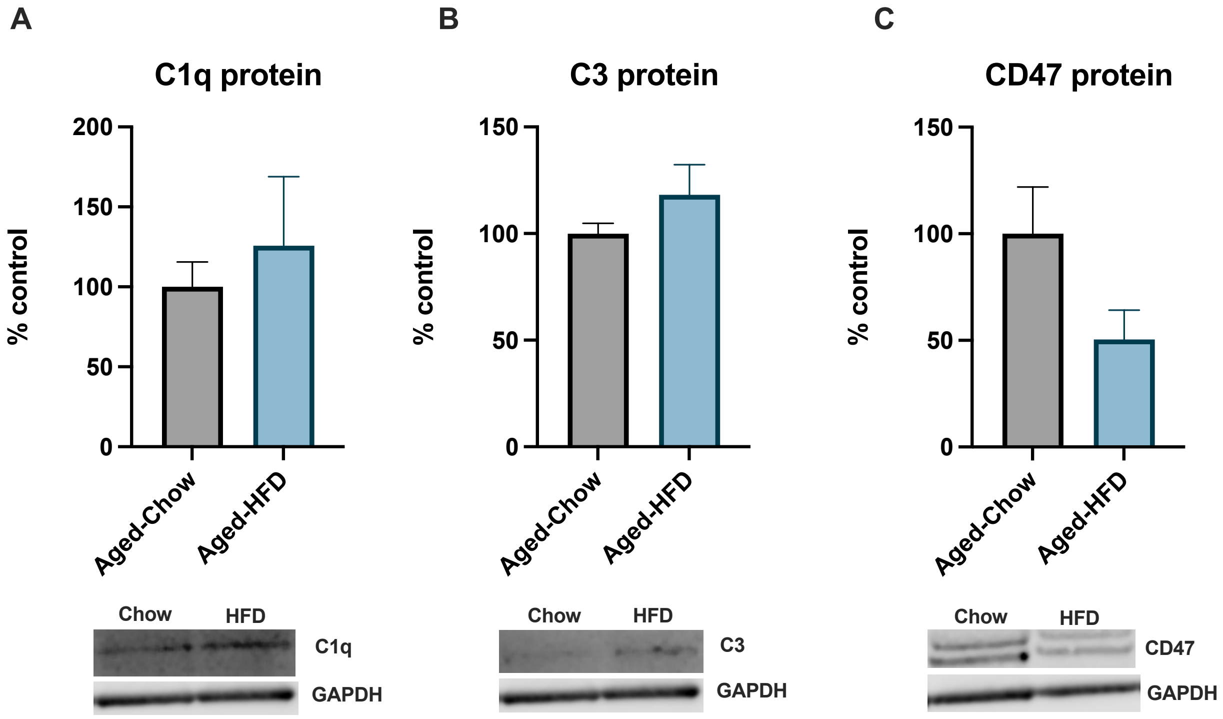

Supplemental Figure 1. HFD modestly increases complement protein concentration and decreases CD47 concentration at the synapse of aged mice. (A) C1q, (B) C3, and (C) CD47.
